# Dinuclear Nickel(I) and Palladium(I) Complexes for Highly Active Transformations of Organic Compounds

**DOI:** 10.3390/molecules23010140

**Published:** 2018-01-11

**Authors:** Takahiro Inatomi, Yuji Koga, Kouki Matsubara

**Affiliations:** Fukuoka University, 8-19-1 Nanakuma, Fukuoka 814-0180, Japan; sd162501@cis.fukuoka-u.ac.jp (T.I.); y-koga@fukuoka-u.ac.jp (Y.K.)

**Keywords:** monovalent nickel, monovalent palladium, dinuclear complexes, catalytic process, DFT calculations

## Abstract

In typical catalytic organic transformations, transition metals in catalytically active complexes are present in their most stable valence states, such as palladium(0) and (II). However, some dimeric monovalent metal complexes can be stabilized by auxiliary ligands to form diamagnetic compounds with metal–metal bonding interactions. These diamagnetic compounds can act as catalysts while retaining their dimeric forms, split homolytically or heterolytically into monomeric forms, which usually have high activity, or in contrast, become completely deactivated as catalysts. Recently, many studies using group 10 metal complexes containing nickel and palladium have demonstrated that under specific conditions, the active forms of these catalyst precursors are not mononuclear zerovalent complexes, but instead dinuclear monovalent metal complexes. In this mini-review, we have surveyed the preparation, reactivity, and the catalytic processes of dinuclear nickel(I) and palladium(I) complexes, focusing on mechanistic insights into the precatalyst activation systems and the structure and behavior of nickel and palladium intermediates.

## 1. Introduction

Interest in the development of catalytic organic transformations using well-defined palladium and nickel complexes as active catalysts continues to increase [1,2,3,4,5,6,7,8,9,10]. Catalytic transformations using these catalysts, such as addition, polymerization, allylic substitution, cross coupling, and C–H substitution reactions, significantly contribute to the production of useful organic chemicals in industry. Interestingly, several dinuclear Pd(I) and Ni(I) complexes have been reported to demonstrate notably higher activity as precatalysts compared to typical monomeric zerovalent or divalent metal complexes [11,12,13,14,15,16]. Detailed mechanistic studies revealed that dinuclear or mononuclear active catalysts are generated from the corresponding dinuclear precatalysts to achieve highly efficient and/or selective catalytic reactions. Although cooperative activation of organic compounds by bimetallic complexes has been reported in organometallic reactions of cluster compounds [17,18,19,20], the mechanism by which dinickel(I) or dipalladium(I) complexes act in catalytic transformations is poorly understood. For example, the in situ formation of dinuclear complexes has been reported to lead to the deactivation of catalytic pathways of highly active mononuclear catalysts. Several other reports proposed that heterolytic or homolytic cleavage of the metal–metal bond of dinuclear Pd(I) or Ni(I) complexes could produce active metal species in situ, such as highly unsaturated “mono-ligated metal complexes”. However, the chemistries of dinuclear Ni(I) and Pd(I) complexes in catalysis have not been classified and compared with each other, although the chemistry of such nickel and palladium complexes has been summarized individually [11,12,13,14,15,16]. Therefore, the survey and comparison of the reactivity and catalytic performance of dinuclear nickel(I) and palladium(I) complexes, as well as their generation processes and structures, should be useful and informative. In this review, the structural features of dinuclear Pd(I) and Ni(I) complexes are discussed, and their catalytic applications and mechanistic details are surveyed in order to uncover the key points of their chemistry.

## 2. Preparation, Structure, and Properties of Dinuclear Nickel(I) and Palladium(I) Complexes

**Chart 1:**
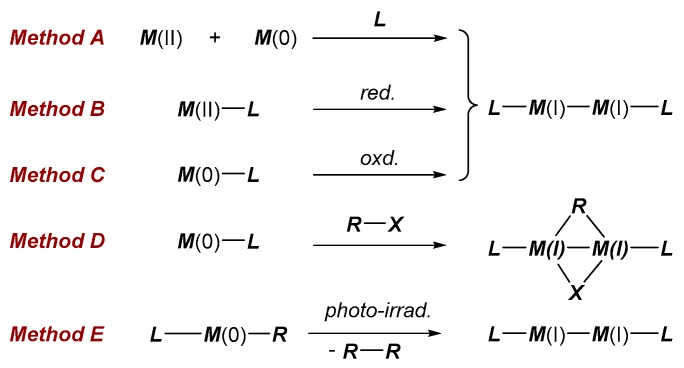
Various methods (Methods A–E) to access dinuclear monovalent complexes of Ni and Pd.

Numerous examples of dinuclear nickel(I) and palladium(I) complexes have been reported in the past decades [11,12,13,14,15,16]. Most of these dinuclear complexes have been efficiently synthesized using several established methods. One of the most accessible routes to these compounds is the treatment of nickel(II) or palladium(II) complexes with the corresponding zerovalent metal complex in the presence of bridging bidentate or tridentate ligands (Method A in Chart 1). Other methods to obtain such dinuclear complexes include 1e reduction of mononuclear divalent complexes (Method B), the oxidation of zerovalent complexes (Method C), and the oxidative addition to zerovalent complexes to form unsaturated divalent complexes, which are stabilized by forming a metal–metal bonding interaction with a second zerovalent complex (Method D). Rare examples of the photoirradiation of alkylmetal complexes, in which the carbon–metal bond was homolytically broken and smoothly dimerized from the unstable monomeric metal radical to form a metal–metal bond (Method E), have also been shown. With multiple routes to access such complexes, the use of dinuclear Pd(I) or Ni(I) complexes as catalyst precursors is increasingly reported, because these compounds show remarkable activities with respect to catalytic transformations.

**Figure 1:**
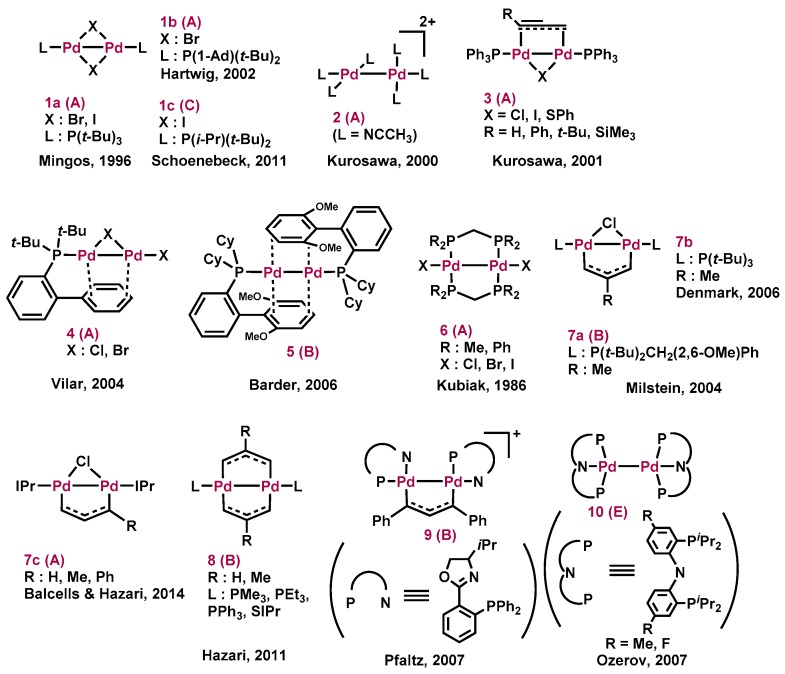
Representative examples of dinuclear Pd complexes. In parenthesis, the synthetic methods A–E are shown.

In Figure 1, representative examples of dinuclear palladium(I) complexes are shown. In 1996, Mingos et al. reported the first example of catalytically active monovalent dipalladium complexes, **1a**. The structure of these complexes was determined by X-ray crystallography, which showed the existence of a Pd(I)–Pd(I) bonding interaction with bridging halogen ligands [21]. The stability of the dinuclear Pd(I) framework in the presence of only halide bridging ligands was discussed recently by Schoenebeck et al. [22]. When tricyclohexylphosphine was used as the ancillary ligand, the halide-bridged dimer did not form. Instead, the use of bis(*t*-butyl)isopropylphosphine, whose steric bulk is intermediate between that of P(*t*-Bu)_3_ and PCy_3_, yielded the stable dimeric complex **1c,** which was similar to Mingos’s dimer.

In the presence of bridging ligands such as monomethylene-bridged bisphosphine, the dinuclear Pd(I) complexes **6** were synthesized and characterized decades earlier than the other complexes, although only reactions with small molecules were investigated [19,23]. Kurosawa and co-workers developed the first example of a series of complexes **3** in which μ:η^3^-allenyl or propargyl ligands bridged the Pd(I)–Pd(I) bond, showing that dipalladium(I) structure was also stabilized by bridging π-ligands. This bond was stable even when smaller phosphine ligands were used instead of P(tBu)_3_ [24]. The complexes were formed by the reaction of Pd(II) allenyl or propargyl complexes with a Pd(0) source. Murahashi and Kurosawa et al. reported a good starting complex for the preparation of various cationic dinuclear Pd(I) complexes via ligand exchange reaction, [Pd_2_(NCCH_3_)_6_]^2+^ (**2**) [25]. For example, this complex was used as a precursor for the bis-dmpm bridged dipalladium(I) dication, [Pd_2_(dmpm)_2_(NCCH_3_)_2_]^2+^, where dmpm is bis(dimethylphosphino)methane.

Vilar et al. successfully developed dipalladium(I) complex **4** using a bulky biphenylphosphine ligand, bis(*tert*-butyl)biphenylphosphine [26]. The complex was obtained by coupling of the Pd(0) phosphine complex with a Pd(II) halide. Complex **4** mediated the Buchwald–Hartwig amination of aryl halides efficiently at room temperature. Barder reported the similar dipalladium(I) complex **5**. This complex was obtained by the reduction of palladium(II) dicyclohexyl(biaryl)phosphine complex with silver tetrafluoroborate salt, leading to the coordination of the μ:η^2^:η^2^-aryl moiety of the biarylphosphine ligand as a bridge between the two unsaturated metals [27]. The existence of 2′,6′-dimethoxy groups on the biaryl substituent was essential to stabilize the dimeric structure. Therefore, the Pd-π interaction strongly stabilized the dipalladium framework.

In 2004, Milstein et al. found that treatment of (2-methylallyl)PdCl_2_ with dmobp (dmobp = di-*tert*-butyl[(2,6-dimethoxyphenyl)methyl]phosphine) in the presence of NaOH yielded the π-allyl-bridged dipalladium(I) complex **7a** [28]. Denmark and Baird later reported the analogous complex **7b**, which used tri-*tert*-butylphosphine instead of the benzylphosphine. This complex has high catalytic activity toward Hiyama cross-coupling reactions of aryl halides with heterocyclic silanolates [29]. Bulky N-heterocyclic carbenes (NHCs) can also be used to stabilize the dinuclear Pd(I) system. Barcells and Hazari et al. synthesized the IPr-supported μ-π-allyl Pd(I) dimers **7c**, which are rare examples with 1-substituted μ-allyl ligands, by the reaction of Nolan’s IPrPd(II)Cl(π-allyl) complex with K_2_CO_3_ in the presence of ethanol [30]. Bis-μ-allyl dipalladium(I) complexes **8** were also prepared by reacting mononuclear bis-π-allyl palladium(II) with free ligand, or by the reaction of μ-allyl palladium(II) chloride with free ligand and the sequential addition of an allyl Grignard reagent. Interestingly, CO_2_ was inserted into the palladium–carbon bond of **8** as an electrophile to form bridging carboxylate complexes, which led to further catalytic processes as described below [12,31].

Pfaltz et al. achieved isolation of an intermediary dipalladium(I) complex **9** using a chelating NP ligand for catalytic nucleophilic substitution of allylbenzoate with malonate [32]. Ozerov et al. successfully synthesized the bridging-ligand-free dipalladium(I) complexes **10** using monoanionic tridentate PNP pincer-type ligands. This was the representative example of a complex without bridging ligands that clearly showed the existence of a Pd(I)-Pd(I) bonding interaction [33] other than Murahashi and Kurosawa’s complex, [Pd_2_(NCCH_3_)_6_]^2+^ (**2**).

Compared to the analogous palladium chemistry, dinuclear nickel(I) complexes and their catalytic applications have been poorly explored. This may be due to the greater stability of ‘monomeric’ monovalent nickel complexes compared to the corresponding palladium(I) complexes. Many examples of monomeric nickel(I) complexes have been reported [34], such as those relevant to the oxygen activation system in bioinorganic chemistry [35]. Additionally, a great difference between the number of studies of the catalytic applications of palladium and nickel can be observed.

**Figure 2:**
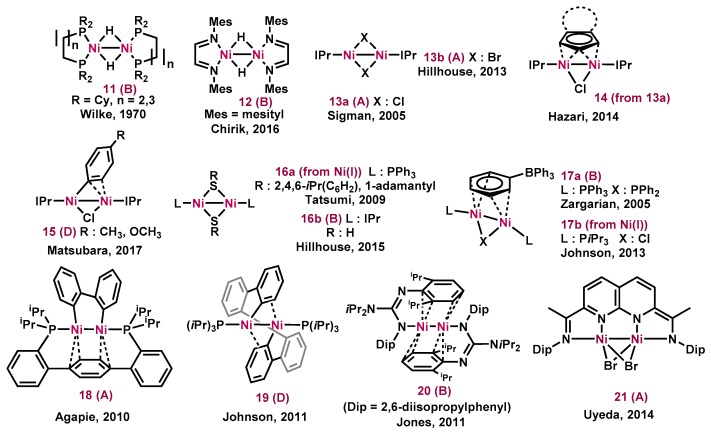
Representative examples of dinuclear Ni(I) complexes. In parenthesis, the synthetic methods A–E are shown.

Initial progress in the chemistry of dinuclear nickel(I) complexes has been promoted by the great interest in unusual interactions of conjugated π-ligands with the dinickel framework, as discussed below [34]. Preparative methods to access the dinickel(I) complexes are quite similar to those of the palladium complexes, as might be expected. Dinickel(I) complexes can be prepared by simply mixing unsaturated monovalent and divalent nickel complexes (Method A), by 1e-oxidation of nickel(0) complexes (Method B), and by oxidative addition on Ni(0) in which the unsaturated adduct is stabilized by forming a metal–metal bonding interaction with a second Ni(0) complex (Method D). Additionally, in these processes, bridging ligands and/or a coordinatively unsaturated site in one of the metal centers lead to the facile formation of the dimeric structures, similar to procedures using bridging aryl, allyl, and cyclopentadienyl groups in palladium chemistry. In Figure 2, representative examples of dinuclear Ni(I) complexes are shown.

In the earliest stage of the development of such complexes, Ni(I) hydride dimers stabilized by bulky chelating bisphosphines (**11**) were reported by Wilke and Jonas [36]. Those complexes were prepared by the reaction of NiCl_2_ (bisphosphine) with NaBMe_3_H, and then obtained via an alternative synthesis from the complex Ni(0) (bisphosphine) (benzene) and H_2_ gas at low temperature [37]. These complexes were thermally quite stable in the solid state and did not decompose to the monomeric form. However, [Ni(dippe)]_2_(μ-H)_2_, (dippe = 1,2-bis(diisopropylphosphino)ethane) reacts readily with cyclohexene sulfide to form sulfur-bridged dimers accompanied by the liberation of H_2_ gas [38], strongly indicating the potential of such Ni(I) hydride dimers as catalyst precursors. Bidentate nitrogen ligands with bulky substituents were also effective as auxiliary ligands in dinuclear Ni(I) complexes. Chirik et al. prepared diamine-supported dinickel(I) hydride complex **12**, which can efficiently mediate the catalytic hydrosilylation of terminal alkenes [39]. 

In 2005, the preparation of chloride-bridged dinickel(I) complex **13a** bearing IPr, where IPr is one of the bulky NHCs, 1,3-bis(2,6-diisopropylphenyl)imidazol-2-ylidene, was reported by Sigman et al. [40]. This complex has a 30e unsaturated nature and is kinetically stabilized by the bulky IPr ligand. Subsequently, analogous dinuclear nickel(I) complexes with other bridging ligands, such as μ-arylimido and alkylene ligands, were produced by Hillhouse et al. through the reaction of Sigman’s dimer with arylazide and diazocarbene reagents [41,42]. These researchers also synthesized the dibromide analogue **13b** [43].

Hazari et al. synthesized a series of dinuclear nickel(I) complexes **14** bearing bridging cyclopentadienyl and indenyl ligands, and compared their reactivity and stability to their palladium analogues [44]. According to this report, decomposition via heterolytic cleavage of the monovalent metal–metal bond to form a pair of zerovalent and divalent metal complexes is unfavorable for dinuclear nickel(I), but likely occurs for dinuclear palladium(I). Moreover, the metal–metal bonds in nickel(I) dimers are more resistant to splitting into monomeric complexes than those of the palladium analogues.

Recently, Matsubara et al. serendipitously found that the reaction of aryl chloride with equal amounts of IPr and Ni (0)(cod)_2_ efficiently formed the dinuclear nickel(I) μ-σ-aryl complex **15** bearing the IPr ligand, rather than the monomeric oxidative adduct [45]. This dinickel(I) complex can also be prepared by the reaction of Sigman’s dimer with 1 equiv. of arylmagnesium chloride via transmetallation. Subsequent reaction with aryl chloride gave the biaryl and regenerated Sigman’s dimer, probably as a result of oxidative addition of aryl chloride and reductive elimination of the biaryl. Therefore, these complexes could potentially be active as catalysts in the Kumada–Tamao–Corriu coupling of aryl halides. Ghadwai et al. attempted to prepare the bromide analogue. However, similar treatment using bromoarenes with Ni(cod)_2_ and IPr provided the 2-arylated imidazolium salt rather than the dinuclear compound, probably due to the lower stability of the bromide analogue [46].

Thiolate is an effective bridging ligand that binds more strongly to metal centers than halide ligands. Tatsumi et al. reported that substitution of the bridging halide ligands with sulfide anions resulted in efficient formation of the corresponding μ-sulfide Ni(I) dimer **16a** [47]. Olechnowicz et al. also synthesized the NHC analogue **16b**, in which the bridging thiolate was μ–SH, from Sigman’s dimer [48]. Interestingly, this complex was also obtained from the heterolytic cleavage of H_2_ in the presence of the sulfido-bridged Ni(II) dimer [Ni(IPr)(μ-S)]_2_ [49]. In addition to the H–H bond, the H–B bond of pinacolborane was also heterolytically cleaved on the dinuclear nickel centers.

Zargarian et al. reported that the decomposition of a divalent nickel complex after anion exchange with a non-coordinating anion such as tetraphenylborate provided the dimeric nickel(I) complex **17a** in which two nickel centers were bridged by a μ-phosphide ligand [50]. Interestingly, one of the phenyl groups bound to borane also bridged the two nickel centers. Similar μ-phenyl bridging coordination was achieved more simply by Johnson et al. [51]. One of the halide ligands on a monovalent nickel complex was exchanged for tetraphenylborate to form an unsaturated nickel(I) center, which combined with another nickel(I) complex to yield dinuclear nickel(I) complex **17b**, which features μ-phenyl bridging coordination by tetraphenylborate.

Agapie et al. reported an interesting finding for the well-defined dinuclear nickel(I) complex **18**, which is stabilized by a terphenylene-bridged diphosphine ligand that reacts with biphenyldimagnesium halide to form a transmetalated μ-biphenylene complex. This complex coupled a pair of biphenylene molecules on the two metal centers to form a bridging C–C bond to give tetraphenylene [52]. The complex also formed a fluorene molecule from biphenylene and dichloromethane. Johnson et al. reported that treatment of biphenylene with a monomeric Ni (0) complex resulted in the oxidative addition of biphenylene to form dinuclear bis(biphenyl) complex **19** containing a Ni(III)–Ni(I) bond [53]. Subsequent reductive coupling of the C–C bond provided a dinuclear tetraphenylene nickel(I) complex, which produced tetraphenylene catalytically in the presence of biphenylene upon heating, accompanied by regeneration of the dinuclear bis(biphenyl) complex. These results indicated that the unsaturated dinickel(I) system can also be effective for catalytic reactions involving concerted activation of substrates by a pair of active metal centers.

π-Coordination bridging by a benzene ring can also stabilize the dinickel(I) framework, as seen in palladium complex **5**. Jones et al. reported that a chelating three-atom-centered guanidinate ligand bearing a bulky DIP substituent (DIP = 2,6-diisopropylphenyl) coordinated to the Ni(I) center in an η^1^ fashion through the nitrogen atom, while one of the aromatic DIP substituents bridged the two Ni(I) centers to stabilize the Ni(I) dimer **20** [54].

In 2014, Uyeda et al. developed redox-active naphthyridine diimine-supported dinickel complexes [55]. Dinuclear Ni(I) complex **21** was formed upon comproportionation of Ni(cod)_2_ and NiBr_2_ in the presence of the naphthyridine diamine ligand. Notably, because of its relatively weak ligand field, the dinuclear Ni(I) complex was paramagnetic, S = 1, in contrast to the diamagnetic phosphine and NHC complexes. Moreover, oxidation and reduction were both found to be possible during electrochemical analysis, and reaction with oxidizing and reducing agents allowed the formation of a series of complexes in different 5e states. The authors also reported several catalytic reactions using the dinickel complex bearing naphthyridine diimine, which retained its dinuclear framework during catalysis [56].

## 3. Catalytic Applications

**Scheme 1:**
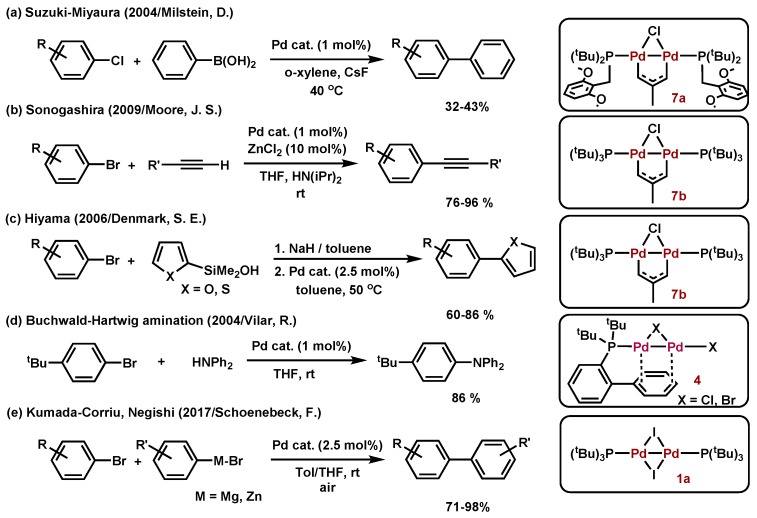
Representative examples of catalytic processes using Pd(I)–Pd(I) complexes.

Many reports of catalytic processes mediated by dinuclear palladium(I) complexes used as catalyst precursors have been published. Notably, almost all of these studies reported remarkably high efficiencies for the catalytic reactions, which included Suzuki–Miyaura coupling [28], Sonogashira coupling [57], Hiyama coupling [29], Buchwald–Hartwig amination [26], Kumada–Corriu and Negishi coupling [58] (Scheme 1), and others [59,60,61], suggesting that dinuclear Pd(I) complexes can generate species with unusually high activity as key intermediates. However, in all but a few examples, it was unclear how these reactions proceeded from the dinuclear Pd(I) complexes. Additionally, there are several examples of catalytic transformations of organic compounds mediated by dinuclear nickel(I) complexes among the huge number of reported catalytic reactions using nickel complexes. The mechanism of these catalytic reactions has been studied in detail. This section focuses on the roles of Pd and Ni bimetallic complexes in catalysis. Several factors, such as the choice of bridging and/or auxiliary ligands, the type of reaction, and the metal center, critically affected these roles. Some of the transformations were activated via the formation of mononuclear mono-ligated, unsaturated metal complexes after disproportionation. In such complexes, re-formation of the dinuclear complex by comproportionation deactivated the complexes, or served to stabilize active form. In other transformations, the intact dinuclear complex activated the substrates efficiently.

### 3.1. Catalysis Using Palladium Complexes

**Scheme 2:**
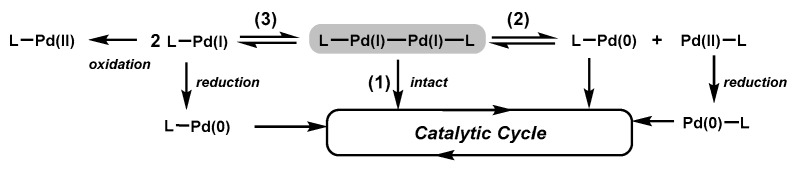
Three possible activation pathways for dinuclear Pd(I) complexes in catalysis: (1) activation of substrates by an intact dimer; (2) disproportionation of a dimer into Pd(0) and Pd(II); and (3) splitting of the dimer into two molecules of Pd(I).

Several possible mechanisms for catalytic transformation by dinuclear palladium(I) complexes have been proposed: (1) Activation of substrates by the intact bimetallic complex; (2) disproportionation of the complex into Pd(0) and Pd(II), which are involved in the catalytic cycle; and (3) homolytic splitting of the complex into two Pd(I) molecules, which are smoothly reduced to Pd(0) or oxidized to Pd(II) (Scheme 2). In the case of (2), an equilibrium between the regenerated dinuclear complex, which exists as a deactivated intermediate, and the active mononuclear complex may sometimes occur [62]. The most probable mechanism is the formation of highly active mono-ligated Pd(0) complexes from the corresponding dimer via mechanism (2) or (3). When monomeric complexes bearing more than two auxiliary ligands are used as catalyst precursors, liberation of these ligands requires harsh conditions. On the other hand, the Pd(I)–Pd(I) bond in dinuclear complexes can be broken quite easily to form the active mono-ligated complex without breaking the stronger metal–ligand bonds. This is probably the mechanism by which dinuclear Pd(I) complexes are active in these catalytic systems. The activation of substrates by unsaturated Pd(I) dimers that do not undergo splitting into mononuclear complexes is rare in palladium chemistry. However, concerted bimetallic activation of the substrates by two adjacent metal centers could possibly induce highly active and/or selective transformations in catalysis. Therefore, new reactions can be developed by designing bimetallic activation processes.

#### 3.1.1. Monomeric Palladium Catalysts from Dinuclear Complexes

In 2002, Hartwig et al. reported the achievement of efficient cross-coupling reactions, the Suzuki–Miyaura coupling and the Buchwald–Hartwig amination, of aryl chlorides at room temperature using the dinuclear palladium(I) complex [Pd(PtBu_3_)]_2_(μ-X)_2_ (X = Cl, Br) (**1b**) [63]. The oxidative addition of aryl chloride was revealed to be the rate-determining step, and the addition of PtBu_3_ to a palladium(0) precursor showed similar activity toward the cross-coupling reactions. Therefore, it was proposed that disproportionation of the dinuclear Pd(I) complex gave the active mononuclear Pd(0) intermediate [Pd(0)(PtBu_3_)], which also could be formed by the reaction of the palladium(0) precursor and PtBu_3_.

**Scheme 3:**
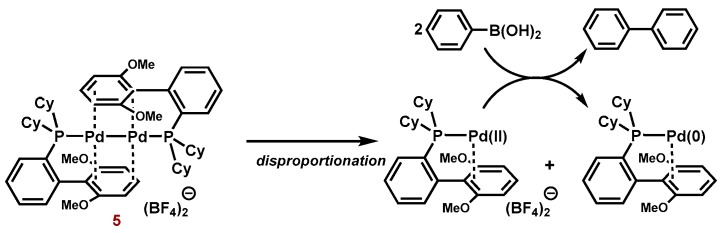
Proposed disproportionation pathway for the initiation of Suzuki–Miyaura coupling.

Barder showed that Suzuki–Miyaura coupling of aryl chloride could be accomplished using cationic palladium(I) dimer **5** bearing a biarylphosphine ligand, in which the π-electrons of the dimethoxy-substituted aryl moieties stabilize the unsaturated palladium centers [27]. Because the dimer complex did not activate aryl chloride at low temperature, it was proposed that formation of active monomeric complex at a temperature above 60 °C may be necessary (Scheme 3). Disproportionation of the bimetallic Pd(I) complex gives not only Pd(0) but also Pd(II) complexes, which could be easily reduced to Pd(0) by transmetallation with arylboronic acid to form biphenyl as a by-product.

The Pd(I) dimer [Pd(IPr)]_2_(μ-Cl)(μ-π-allyl) (**7c**) was detected and determined to be a reaction intermediate in the Suzuki–Miyaura coupling of aryl chloride in the presence of [Pd(II)(IPr)(π-allyl)Cl] and a base by Balcells and Hazari et al. [30]. The dimer complex **7c** can easily disproportionate into the monomeric complexes IPr-Pd(0) and Pd(II) π-allyl to initiate the catalytic process; moreover, addition of an equivalent of [Pd(II)(IPr)(π-allyl)Cl] was found to deactivate the process. Therefore, a comproportionation process producing an equilibrium between the Pd(I) π–allyl dimer and the mononuclear complexes was also identified, demonstrating that the Pd(I) dimer **7c** forms as an off-cycle product. Several studies identifying a dinuclear Pd(I) complex as the resting state outside of the catalytic cycle have also been reported [64,65,66,67]. 

**Scheme 4:**
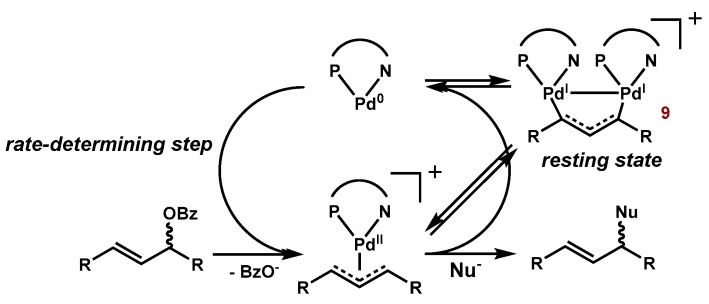
Proposed mechanism of Pd-catalyzed allylic substitution.

Pfaltz et al. reported the palladium-catalyzed nucleophilic substitution of allylbenzoate with malonate, an example of the formation of a dinuclear structure proposed to be an off-cycle product that stabilized the active mononuclear intermediates [32]. They found a reversible pathway from the mononuclear Pd(0) and Pd(II) π-allyl complexes to the dinuclear Pd(I) complex **9**, in which a π-allyl moiety bridged the two metal centers. However, further reaction of the dinuclear complex with malonate did not yield the substitution product, indicating that the dimer is not active in the catalytic transformation. This dinuclear Pd(I) complex reversibly produced the mononuclear Pd(II) π-allyl complex in the presence of allylbenzoate (Scheme 4).

**Scheme 5:**
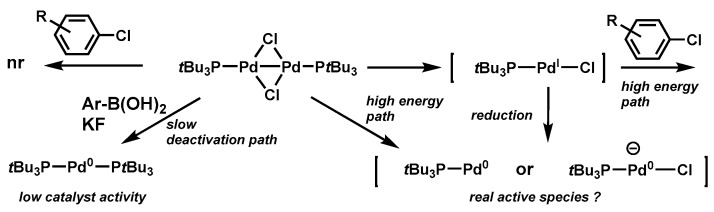
Possible pathways for the activation of the Pd(I) dimer.

Schoenebeck et al. reported that the initiation process of the Heck reaction did not involve simple heterolytic dissociation of the dinuclear palladium(I) complex [Pd(PtBu_3_)]_2_(μ-Cl)_2_ to form the corresponding mono-ligated Pd(0) active catalyst [68]. Instead, formation of the monomeric Pd(I) complex [Pd(PtBu_3_)Cl] by homolytic cleavage of the Pd(I) dimer is more favorable, as it proceeds with a much lower free energy (ΔG_diss_ = 24.8 kcal mol^−1^) than the heterolytic formation of [Pd(0)(PtBu_3_)] and [Pd(II)(PtBu_3_)Cl_2_] (ΔG_diss_ = 38.1 kcal mol^−1^), as estimated by DFT calculations. Additionally, the dimer complex itself, as well as its decomposition product Pd(0)(PtBu_3_)_2_, which was gradually generated from the dimer in the presence of aryl boronic acid and KF, were revealed to be less active toward catalysis. Therefore, these authors proposed that the true active catalyst was most likely to be the mono-ligated or anionic complex, [Pd(0)(PtBu_3_)] or [Pd(0)(PtBu_3_)Cl]^-^, formed by reduction of the monomeric Pd(I) intermediate [Pd(PtBu_3_)Cl] (Scheme 5).

#### 3.1.2. Proposed Mechanisms Involving Dinuclear Palladium Catalysts

The possibility of catalytic processes involving an active dipalladium(I) complex that retains its dinuclear framework has also been proposed. In 2010, Bera et al. reported that a dinuclear Pd(I) complex bearing tridentate 1,8-naphthyridine derivative ligands mediated Suzuki–Miyaura cross coupling of aryl halides [69]. Although 3-bromopyridine was effective for cross coupling, 2-bromopyridine was not suitable for the coupling reaction, and yielded only the starting material. They therefore proposed the presence of a metal coordination site adjacent to the active metal center that could be easily bridged by 2-bromopyridine in the oxidative addition process. However, definitive identification of the bimetallic system in catalysis would require more detailed study.

**Scheme 6:**
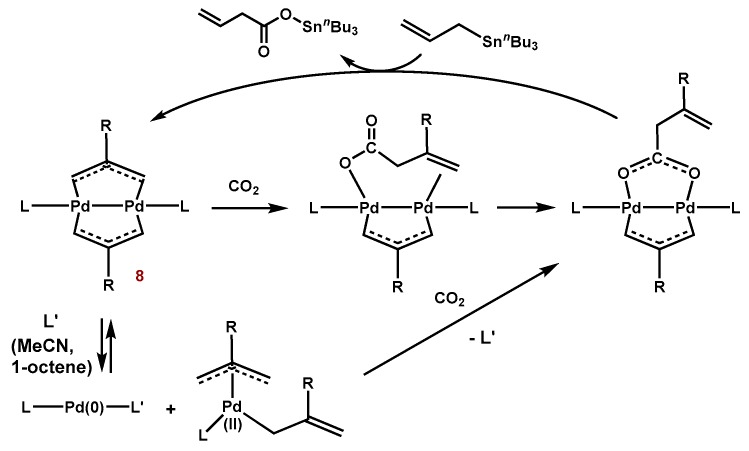
Proposed catalytic cycle of allylic carboxylation using a bis(μ-π-allyl)dipalladium(I) complex, which is supported by experimental results.

Subsequently Hazari et al. proposed that an intact dinuclear palladium(I) complex acts as a bimetallic catalyst in the allylic carboxylation of allylstannane and allylboronate with CO_2_ [31]. They found that stoichiometric reaction of the bis-μ-π-allyl Pd(I) complex with CO_2_ afforded a bridged mono-carboxylate dipalladium(I) complex via facile insertion of CO_2_ into the palladium-π-allyl bond. Subsequent addition of allylstannane and allylboronate gave the starting π-allyl complex accompanied by liberation of allylcarboxylate (Scheme 6). Detailed theoretical calculations also indicated that the dinuclear framework of the dipalladium(I) catalyst remains intact during the catalytic allylic carboxylation [70]. However, the addition of weakly coordinating ligands, such as acetonitrile and 1-octene, accelerated the catalytic reaction. Moreover, the further addition of these ligands or the addition of other ligands yielded mononuclear Pd(0) complexes and palladium black [71]. Therefore, another catalytic pathway involving insertion of CO_2_ into a mononuclear Pd(II) complex was proposed. In this pathway, coordination of the ligand L’ stabilizes the disproportionated mononuclear Pd(0) complex in an equilibrium with the dinuclear complex, and the accompanying formation of the σ-allyl Pd(II) complex enables the insertion of CO_2_ into the Pd-carbon bond.

**Scheme 7:**
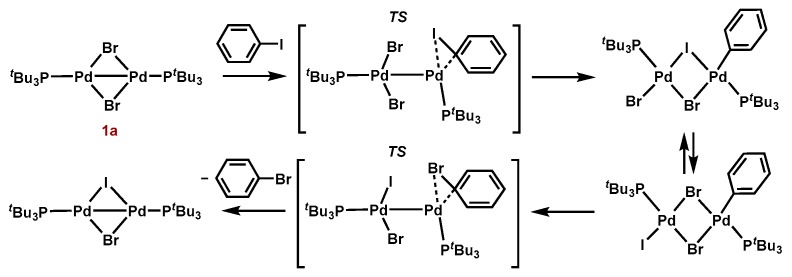
Mechanism of halogen exchange of iodobenzene, proposed based on theoretical studies.

The most remarkable process involving dinuclear Pd(I) complexes is a series of halogen exchange reactions of aryl halides studied by Schoenebeck et al. [72,73]. Interestingly, the reaction was not catalyzed by mononuclear Pd(II) halide. However, when a dinuclear Pd(I) catalyst was used, the two bridging halogen ligands of the complex underwent stepwise exchange, as observed using ^31^P NMR spectroscopy. A detailed theoretical study revealed that the oxidative addition of aryl iodide occurs at a single metal center that retains its dinuclear framework. After the site exchange process of the halogen ligand on the Pd(II) dimer, the semi-stable dipalladium(II) adduct intermediate eliminates aryl bromide to generate the Pd(I) dimer (Scheme 7). A similar reaction mechanism was proposed for the halogen exchange reaction of aryl iodide with ^-^SCF_3_ or SeCF_3_ [74,75].

The chemistry of Pd(I) dimer in catalysis has been summarized as follows. The above studies on catalytic systems mediated by dinuclear Pd(I) complexes revealed that the formation of monomeric Pd(0) and/or Pd(II) complexes as the true active catalytic species is the key process to initiate most reactions. Coordinatively unsaturated, mono-ligated palladium complexes of the form Pd(0)L are proposed to be very active catalysts that enable facile activation of substrates, whereas typical Pd(0)L_2_ complexes are less active for these reactions. Several pathways to generate the monomeric complexes can be proposed, namely, the simple disproportionation of the dimer into zerovalent and divalent complexes, and the reduction or oxidation of Pd(I) complexes produced by homolytic cleavage of the Pd(I)–Pd(I) bond. Additionally, in situ generated, inactive dinuclear Pd(I) complexes sometimes stabilize the unstable monomeric active species as resting states, and it is quite rare for intact dinuclear complexes to act as catalysts. It has also been reported that only one of the metal centers directly activate the C–X bond of aryl halide in the oxidative addition process.

In many cases, it is very difficult to clarify whether the active compound is a mononuclear or dinuclear complex. However, theoretical calculations combined with experimental results can suggest possible pathways. These results reveal the nature of dinuclear Pd(I) complexes and new possible transformations using these complexes, thus enabling progress in the development of highly efficient reactions using bimetallic palladium systems in the near future.

### 3.2. Catalysis Using Nickel Complexes

**Scheme 8:**
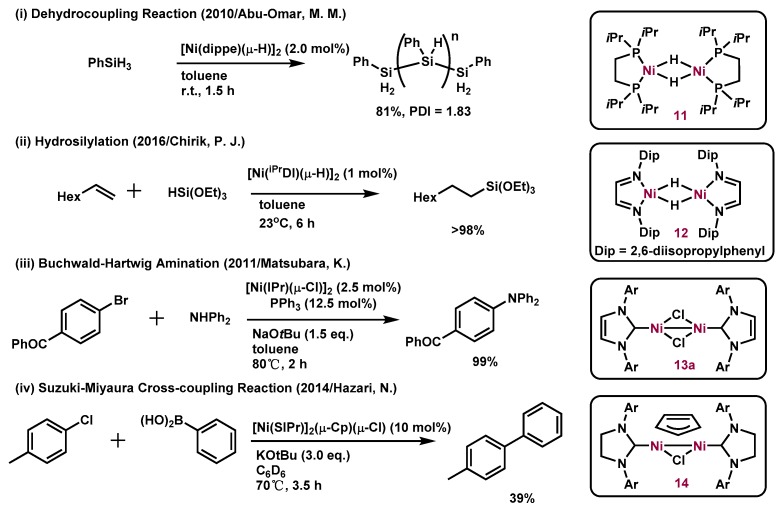
Examples of catalytic processes using dinuclear Ni(I) catalyst precursors.

**Scheme 9:**
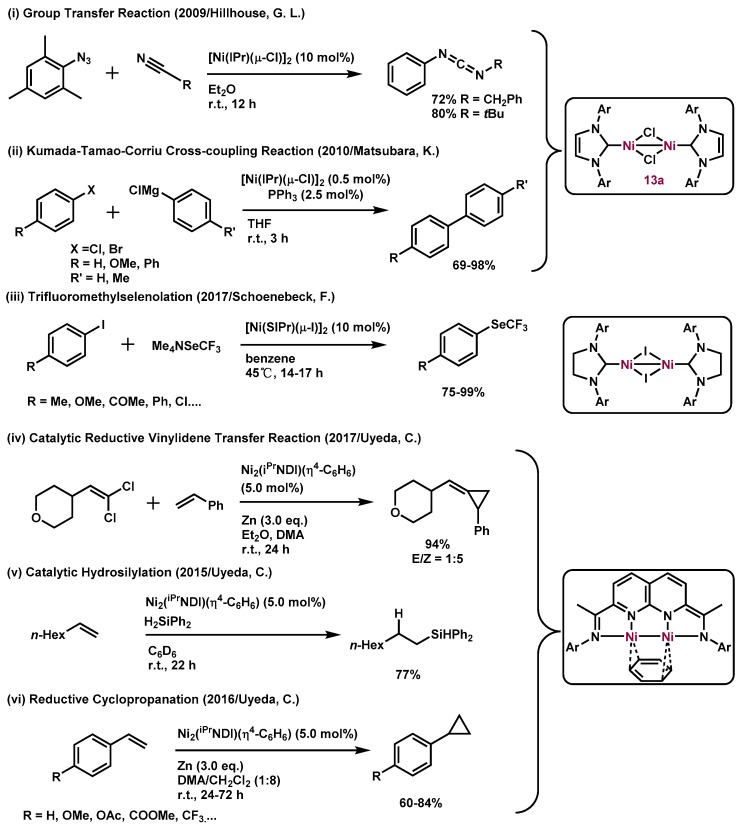
Examples of catalytic processes using dinuclear Ni complexes, in which the complexes were proposed to retain their dinuclear frameworks.

As noted above, studies on catalytic transformations using dinuclear nickel(I) complexes have been less reported compared with the corresponding palladium chemistry (Scheme 8 and Scheme 9). The studies that have been performed using nickel(I) complexes indicated the catalytic reactions were initiated by similar processes: Homolysis or heterolysis of Ni(I)–Ni(I) bonds to form mononuclear active catalysts (Scheme 8), and catalysis by intact dinuclear complexes (Scheme 9). When the dinuclear structures remain intact during catalysis, catalytic transformations using dinuclear nickel(I) complexes can proceed similarly to those using palladium. On the other hand, the greatest difference in the chemistry of nickel is that its paramagnetic, monovalent mononuclear complexes are much more stable than the corresponding palladium complexes. Therefore, homolytic cleavage of the Ni(I)–Ni(I) bond occurs readily to form the coordinatively unsaturated Ni(I) complexes, which are anticipated to have high catalytic activity.

#### 3.2.1. Mononuclear Nickel Catalysts from Dinuclear Complexes

**Scheme 10:**
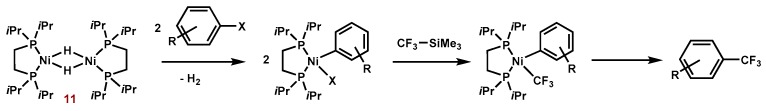
Stoichiometric reactions of dinuclear Ni(I) hydride complexes.

In contrast to the chemistry of palladium, there have been few reports of mononuclear Ni complexes generated from the corresponding Ni(I) dimers playing a key role in catalysis. Some catalytic reactions involving dinuclear Ni(I) hydride complexes have been studied, along with their mechanisms, and mononuclear active nickel catalysts were proposed. Abu-Omar reported the catalytic dehydrocoupling of organohydrosilanes, and also isolated the mononuclear Ni(II) silyl complex formed by the reaction of [Ni(dippe)(μ-H)]_2_ (**11**) (dippe = 1,2-diisopropylphosphinoethane) with HSiCl_3_ [76]. Interestingly, the dinickel hydride complex reacts with chlorobenzene to form a Ni(II) oxidative addition product, via generation of 2 equiv. of Ni(0) accompanied by evolution of hydrogen gas. Vicic et al. also performed a stoichiometric reaction using Ni(0) complexes generated from the same Ni(I) hydride complex (**11**), involving the oxidative addition of aryl halides, transmetallation with alkylsilane, and reductive elimination of alkylarene (Scheme 10) [77]. Therefore, Ni(I) hydride dimers are a good precursor to generate highly active Ni(0) catalysts in situ.

**Scheme 11:**
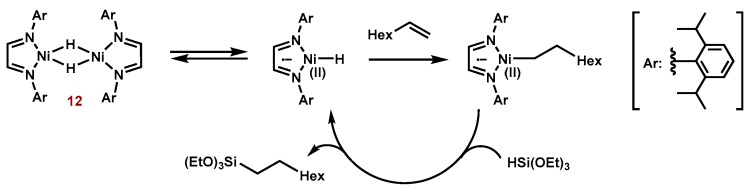
Proposed mechanism for the hydrosilylation of 1-octene with HSi(OEt)_3_. The α-diimine ligands are redox-active, and 1e reduction of the α-diimine forms Ni(II) centers.

Chirik et al. reported the use of the α-diimine-chelated nickel(I) hydride dimer **12** as a catalyst precursor for the efficient catalytic hydrosilylation of terminal aliphatic alkenes in an anti-Markovnikov manner [39]. Interestingly, in contrast to the electronic structure of the diphosphine analogue, the dimer was represented as a complex with two Ni(II) centers with two 1e reduced α-diimine ligands. Therefore, the dimer was proposed to form mononuclear Ni(II) monohydride. DFT calculations indicated that smooth insertion of the alkene into the Ni–H bond can form monomeric Ni(II) alkyl complex (Scheme 11).

**Scheme 12:**
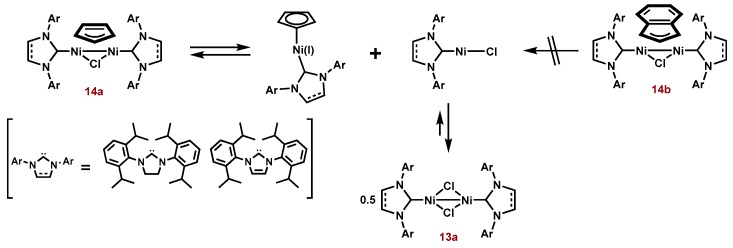
Thermal equilibrium between the dinuclear Ni(I) Cp complex and the catalytically active mononuclear Ni(I) Cp complex. This equilibrium does not occur for the indenyl analogue.

Hazari et al. reported the synthesis of dinuclear Ni(I) complexes bearing bridging cyclopentadienyl and indenyl ligands, [Ni(NHC)]_2_(μ-Cl)(μ-Cp) (**14a**) and [Ni(NHC)]_2_(μ-Cl)(μ-Ind) (**14b**) (Cp = cyclopentadienyl and Ind = indenyl) [44]. The dinuclear Cp complex **14a** undergoes homolytic Ni–Ni bond cleavage and exists in an equilibrium between mononuclear Ni(NHC)(η^5^-Cp) and the dichloride Sigman’s dimer **13a** at room temperature; however, a similar equilibrium was not observed for the bridging indenyl analogue **14b**. This suggests that the Cp ligand stabilizes the mononuclear Ni(I), but the indenyl ligand does not (Scheme 12). Interestingly, the Cp complexes mediated the Suzuki–Miyaura cross coupling of 4-chlorotoluene, but the dinuclear indenyl complex did not, strongly suggesting that only the mononuclear Ni(I) complex has activity toward this catalytic transformation.

**Scheme 13:**
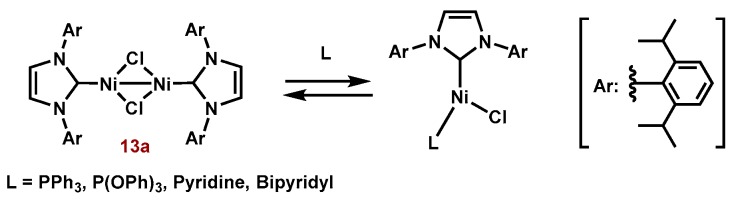
Equilibrium between dinuclear and mononuclear Ni(I) complexes in the presence of a ligand (L). Both complexes are active for cross-coupling reactions.

Matsubara et al. found that Sigman’s dimer **13a** is in equilibrium with the stable mononuclear Ni(I) complex in the presence of an appropriate ligand, such as phosphine, phosphite, or pyridine (Scheme 13) [78,79]. They also reported catalytic reactions using these mononuclear complexes in the presence of an excess amount of the ligands, such as Kumada–Tamao–Corriu cross coupling, Suzuki–Miyaura cross coupling, and Buchwald–Hartwig amination of aryl halide. Notably, the mononuclear Ni(I) complexes bearing a bulky NHC were thermally stable even in the absence of a redox-active ligand.

#### 3.2.2. Proposed Mechanisms Involving Dinuclear Nickel Catalysts

**Scheme 14:**
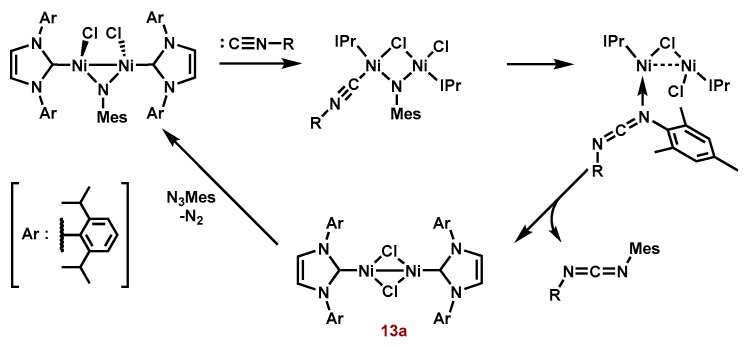
Proposed mechanism for the catalytic nitrene transfer reaction of azide, based on stoichiometric reactions.

In 2009, Hillhouse et al. reported a dinuclear Ni(I) complex bearing a bridging imide ligand as a product of the reaction of Sigman’s dimer, [Ni(IPr)(μ-Cl)]_2_, with mesityl azide (N_3_Mes). Subsequently, the coupling reaction of the imide ligand with isonitrile gave isocyanate derivatives accompanied by the regeneration of the starting Sigman’s dimer (Scheme 14) [41]. This reaction sequence can be applied to catalytic nitrene transfer reactions of N_3_Mes and isonitrile. It was unclear whether mononuclear species were generated or not in this reaction sequence.

**Scheme 15:**
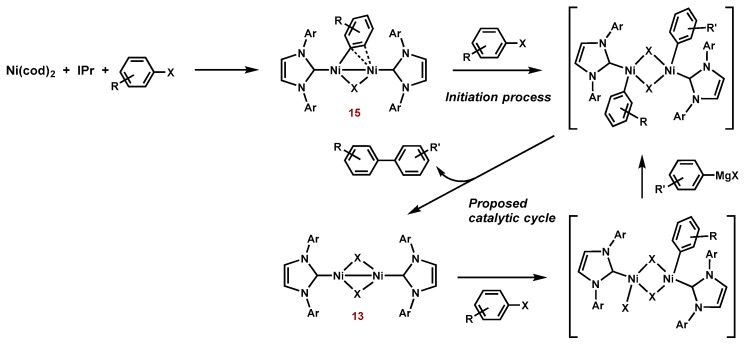
Proposed mechanism of the Kumada–Tamao–Corriu coupling of aryl halide on a dinuclear Ni(I) system, supported by DFT calculations.

**Scheme 16:**
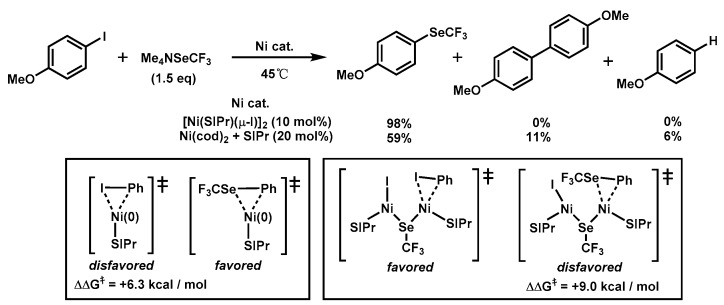
Selectivity divergence between Ni(0) and Ni(I)–Ni(I) in the trifluoromethylselenolation of aryl iodide. The calculated transition states for the oxidative addition of the Ph–X bond to Ni(0) and Ni(I)–Ni(I) showed different favored substituents X, supporting the chemoselectivity of the catalysis.

Recently, Matsubara et al. reported that Sigman’s dimer **13a** acts as a highly efficient catalyst in the Kumada–Tamao–Corriu cross coupling of aryl halides [45]. Generally, a Ni(II) oxidative addition product is obtained from the reaction of a Ni(0) precursor with aryl chloride. However, the reaction of aryl chloride with Ni(0)(cod)_2_ in the presence of 1 equiv. of the bulky IPr ligand unexpectedly yielded the dinuclear Ni(I) adduct **15** rather than mononuclear Ni(II) (Scheme 16). This dinuclear Ni(I) μ-σ-aryl complex **15** was also active in the Kumada coupling, and can alternatively be prepared by the reaction of Sigman’s dimer **13a** with phenylmagnesium chloride via transmetallation. Further stoichiometric reaction of the μ-σ-aryl complex **15** with aryl chloride afforded the biaryl as the cross-coupling product, accompanied by regeneration of Sigman’s dimer **13a**. Therefore, the authors suspected that both dinuclear Ni(I) complexes were involved in the catalytic cycle. However, because the distribution of the biaryl products from the dinickel(I) μ-σ-aryl complex in the stoichiometric reaction was not consistent with that in the catalysis, an alternative pathway involving the oxidative addition of aryl halide to Sigman’s dimer **13a** was proposed (Scheme 15), in which the μ-σ-aryl complex **15** could act as a precursor in catalysis. DFT calculations indicated that the addition of chlorobenzene to the dimer occurs without loss of the dinuclear framework, resulting in a semi-stable dinuclear Ni(II) complex similar to that reported for the palladium analogue [72]. Subsequent transmetallation with phenylmagnesium chloride forms the biaryl and Sigman’s dimer. Notably, the Ni(II)–Ni(0) complex with a dative bond from Ni(0) to Ni(II) is temporally formed just before the oxidative addition to one of the nickel centers to give the Ni(II)–Ni(II) complex. This catalytic reaction afforded no homo-coupling product, probably because the equilibrium of homolysis into mononuclear intermediates was not possible, which prevented the intermolecular aryl exchange process. This fact also strongly supports the existence of the dinuclear system during catalysis.

Schoenebeck et al. also reported a theoretical study on the oxidative addition of iodobenzene to the Ni(I) iodide dimer [Ni(SIPr)]_2_(μ-I)_2_, which is analogous to Sigman’s dimer **13a**. The results indicated that this reaction occurred via a pathway similar to Matsubara’s process [80], involving the efficient catalytic substitution of aryl iodide with Me_4_NSeCF_3_ to yield trifluoromethylselenoarene. Similarly, the biaryl homo-coupling product did not form in this transformation, and no signals were assigned as mononuclear Ni(I) species in the EPR spectra, suggesting that the existence of side reactions involving mononuclear Ni(I) species can be ruled out. The authors isolated the diselenide Ni(I) dimer, [Ni(SIPr)(μ-SeCF_3_)]_2_, as a possible intermediate complex. In contrast to the selectivity of mononuclear Ni(0) catalysts, DFT calculations supported the chemoselectivity of the oxidative addition of aryl iodide rather than aryl selenide to dinuclear Ni(I) (Scheme 16).

**Scheme 17:**
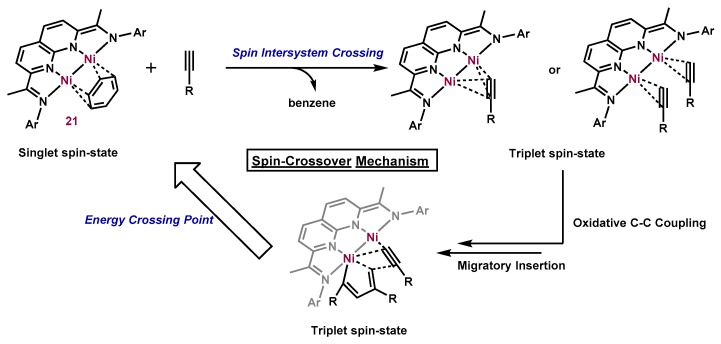
Proposed system for the cyclotrimerization of terminal alkynes via oxidative C–C bond coupling and migratory insertion of the third alkyne on the dinickel(I) centers, which provides chemoselective formation of the benzene derivatives.

In sharp contrast to other dinuclear Ni(I) systems, the dinickel(I) complex reported by Uyeda et al. (*^i^*^Pr^NDI)Ni_2_Br_2_ (**21**) (*^i^*^Pr^NDI = 2,6-diisopropylphenyl-substituted naphthyridine diimine) has two unique features: (1) The positions of the two nickel centers are fixed by the tetradentate π-conjugated auxiliary ligand; and (2) the NDI ligand is redox-active and has two nickel centers, enabling reversible oxidation and reduction with intersystem spin crossing [55]. Moreover, using analogous Ni(0) benzene complex, several catalytic reactions, such as the hydrosilylation and cyclotrimerization of alkynes, were performed on the dinickel centers without breakage of the dinuclear framework. Detailed theoretical studies were conducted by Uyeda and Ess et al. to reveal the mechanism of catalytic alkyne cyclotrimerization (Scheme 17) [81]. Interestingly, oxidative C–C bond coupling of terminal alkynes occurs on the dinickel centers, and migratory insertion of the third alkyne provides chemoselective formation of benzene derivatives, while the reaction occurs via another pathway in the mononuclear catalyst system to provide different product selectivity.

Catalytic reactions using dinuclear nickel(I) complexes are summarized. Although there are very few examples, both mechanisms involving highly active mononuclear complex and intact dinuclear complex as key intermediates are proposed in the efficient catalysis, as in the palladium chemistry. Dinuclear μ-hydride complexes can generate coordinatively unsaturated mononuclear Ni(0) complexes irreversibly as a result of elimination of hydrogen gas, whereas simple homolytic cleavage of the Ni–Ni bond occurs in dinuclear complexes bearing other bridging ligands to form monomeric Ni(I) complexes. Although such mononuclear Ni(I) complexes are proposed as key intermediates in catalysis, it should be noted that direct observation of the Ni(I) complexes as key intermediates has not been reported until now. On the other hand, theoretical calculation is a powerful method to propose the intact dinuclear active complexes involved in the catalytic cycles. Finally, appropriate design of the bridging ligands, naphthyridine diamine, can form dinuclear Ni complex, which has a robust bimetallic framework. A series of catalytic transformations with specific chemoselectivity clearly demonstrated the importance of the chemistry of dinuclear Ni(I) complexes as catalysts.

## 4. Conclusion and Perspectives

In this mini-review, the recently reported structures, catalytic applications, and mechanistic details of dinuclear Pd(I) and Ni(I) complexes are surveyed. As noted in the Introduction, highly efficient catalytic transformations have been achieved under mild conditions using these complexes as catalyst precursors. However, these involve several different activation processes, depending on the metal atoms, ligand structures, and kinds of reactions employed. One of the most interesting points of these activation processes is that pairs of active metal sites were able to activate substrates in a concerted fashion while retaining their dinuclear frameworks. For instance, the oxidative addition of aryl halide to M(I)–M(I) (M = Pd and Ni) generates not the mononuclear M(III) species, but instead the dinuclear adduct, a M(II)-M(II) complex stabilized by bridging ligands. On the other hand, the in situ “reversible” formation of dinuclear complexes may contribute to the stabilization of highly active mononuclear catalysts, which are essential for efficient catalytic transformations. The achievement of a dissociation equilibrium between semi-stable complexes with weak metal–metal bonds that act as “bimetallic dormant species” facilitates the formation of highly active mononuclear complexes without using an excess of ancillary ligands, and moreover, avoids the decomposition of such active complexes.

Although the above chemistry of the dinuclear metal complexes in catalysis has been revealed to have great promise for the development of various useful organic transformations, it is still only at the starting line. The concerted activation of substrates and the development of highly efficient and/or chemoselective catalytic processes show great potential to expand widely in the fields of organic synthesis and applications.
